# PACAP Inhibits β-cell Mass Expansion in a Mouse Model of Type II Diabetes: Persistent Suppressive Effects on Islet Density

**DOI:** 10.3389/fendo.2013.00027

**Published:** 2013-03-11

**Authors:** Hiroaki Inoue, Norihito Shintani, Yusuke Sakurai, Shintaro Higashi, Atsuko Hayata-Takano, Akemichi Baba, Hitoshi Hashimoto

**Affiliations:** ^1^Laboratory of Molecular Neuropharmacology, Graduate School of Pharmaceutical Sciences, Osaka UniversitySuita, Osaka, Japan; ^2^Japan Society for the Promotion of ScienceTokyo, Japan; ^3^Department of Experimental Disease Model, Molecular Research Center for Children’s Mental Development, United Graduate School of Child Development, Osaka University, Kanazawa University, Hamamatsu University School of Medicine, Chiba University and University of FukuiSuita, Osaka, Japan; ^4^School of Pharmacy, Hyogo University of Health SciencesKobe, Hyogo, Japan

**Keywords:** β cells, KKA^y^ mice, high-fat diet, pituitary adenylate cyclase-activating polypeptide, islet neogenesis, type 2 diabetes

## Abstract

Pituitary adenylate cyclase-activating polypeptide (PACAP) is a potent insulinotropic G-protein-coupled receptor ligand, for which morphoregulative roles in pancreatic islets have recently been suggested. Here, we evaluated the effects of pancreatic overexpression of PACAP on morphometric changes of islets in a severe type II diabetes model. Following cross-breeding of obese-diabetic model KKA^y^ mice with mice overexpressing PACAP in their pancreatic β-cells, the resulting KKA^y^ mice with or without PACAP transgene (PACAP/+:A^y^/+ or A^y^/+ mice) were fed with a high-fat diet up to the age of 11 months. Pancreatic sections from 5- to 11-month-old littermates were examined. Histomorphometric analyses revealed significant suppression of islet mass expansion in PACAP/+:A^y^/+ mice compared with A^y^/+ mice at 11 months, but no significant difference between PACAP/+ and +/+ (wild-type) mice, as previously reported. The suppressed islet mass in PACAP/+:A^y^/+ mice was due to a decrease in islet density but not islet size. In addition, the density of tiny islets (<0.001 mm^2^) and of insulin-positive clusters in ductal structures were markedly decreased in PACAP/+:A^y^/+ mice compared with A^y^/+ mice at 5 months of age. In contrast, PACAP overexpression caused no significant effects on the level of aldehyde-fuchsin reagent staining (a measure of β-cell granulation) or the volume and localization of glucagon-positive cells in the pancreas. These results support previously reported inhibitory effects of PACAP on pancreatic islet mass expansion, and suggest it has persistent suppressive effects on pancreatic islet density which may be related with ductal cell-associated islet neogenesis in type II diabetes.

## Introduction

Pituitary adenylate cyclase-activating polypeptide (PACAP) is an extraordinarily potent insulinotropic peptide (Yada et al., 1994) belonging to the vasoactive intestinal polypeptide (VIP)/secretin/glucagon superfamily, which also includes glucagon-like peptide-1 (GLP-1) and glucose-dependent insulinotropic peptide (GIP) (Vaudry et al., 2009). PACAP and its receptors [PACAP-specific PAC1, and VIP-shared VPAC1 and VPAC2 receptors, which belong to the class B (class II) G-protein-coupled receptor family] are highly expressed in neural elements, suggesting that it acts as a neurotransmitter and neuromodulator both in brain and peripheral tissues (Vaudry et al., 2009). There have been numerous studies on PACAP, in which its metabolic effects have been well documented (Ahrén, 2008; Vaudry et al., 2009). For example, PACAP has been shown to reduce food intake, increase glucose uptake in adipocytes by potentiating insulin action, stimulate the secretion of glucagon from the pancreas and norepinephrine from the adrenal medulla, in addition to its insulinotropic activities in the pancreas. Based on these reports, several studies have evaluated the therapeutic potential of agonists or antagonists of the PACAP/VIP receptors (including PACAP and/or VIP themselves), and an inhibitor of dipeptidyl peptidase-4 (DPP-4), a common degradation enzyme for PACAP, VIP, GIP, and GLP-1, for the treatment of metabolic syndrome, including diabetes mellitus (Ahrén, 2008, 2009; Verspohl, 2009; Chapter et al., 2010). DPP-4 inhibitors are currently used as anti-diabetic agents (Holst, 2006; Verspohl, 2009).

As a potential cure for diabetes mellitus, a disease resulting from insulin insufficiency, recent studies have raised the possibility of the enhancement of endogenous β-cell mass, and transplantation of islets themselves, as a novel therapeutic strategy (Vaithilingam et al., 2008; Hanley, 2009; Verspohl, 2009; Dalle et al., 2011). With respect to this possibility, some studies have revealed that PACAP can stimulate β-cell proliferation and suppress the effects of harmful exogenous insults on the β-cell (Yamamoto et al., 2003; Nakata et al., 2010), although it has also been shown that PACAP has inhibitory effects on the pancreatic islet mass (see our review article, Sakurai et al., 2011). Recent studies using animal models in which PACAP/VIP signaling molecules have been knocked out have shown an increased mean islet area in PACAP-KO mice (Tomimoto et al., 2008) and altered islet architecture in VPAC1-KO mice (Fabricius et al., 2011), although no significant defects have been reported in VIP-KO mouse islets (Martin et al., 2010). These results suggest possible roles of PACAP/VIP signaling in islet morphoregulation, although it remains unknown how they regulate islet morphology, particularly in the case of type II diabetes.

Approximately a decade ago, we generated mice either lacking PACAP (Hashimoto et al., 2001) or overexpressing PACAP specifically in pancreatic β-cells (PACAP/+ mice; Yamamoto et al., 2003). To explore the long-term effects of PACAP in type II diabetes, we cross-bred PACAP/+ mice with agouti yellow KKA^y^ mice, an obesity-induced type II diabetic model (Iwatsuka et al., 1970), and showed that pancreatic PACAP overexpression attenuated hyperinsulinemia and islet hyperplasia in KKA^y^ mice, without any alteration of plasma glucose, glucose tolerance, or insulin tolerance (Tomimoto et al., 2004). Since the mild and delayed-onset hyperglycemia in KKA^y^ mice (Srinivasan and Ramarao, 2007) might mask the effects of PACAP in this model, we recently re-examined the phenotypic effects of PACAP overexpression in KKA^y^ mice fed a high-fat diet (HFD) (Sakurai et al., 2012). The results showed that HFD feeding of KKA^y^ mice induced severe, early-onset diabetes, but caused an unexpected recovery from hyperglycemia between 6 and 11 months of age, partly due to simultaneous (6–10 months of age) hyperinsulinemia. We also found that PACAP overexpression retained its previously observed suppressive effects, particularly those relating to hyperinsulinemia, in HFD-fed KKA^y^ mice (Sakurai et al., 2012), however there has been no reported morphological information on the pancreatic islets of this model.

In the present study, we performed several morphometrical analyses of the islet phenotype of HFD-fed KKA^y^ mice, including staining with hematoxylin-eosin (HE), aldehyde-fuchsin (AF), and anti-insulin and anti-glucagon antibodies. Here, we used PACAP/+ mice to evaluate the direct and local action of PACAP on the islet morphology, since PACAP can exert pleiotropic actions on adipocyte and adrenal medulla in addition to islets and secondary affect islets. The results obtained show that PACAP retains its inhibitory effects on pancreatic islet mass expansion, and provide a range of evidence suggesting that pancreatic PACAP affects ductal cell-associated islet neogenesis.

## Materials and Methods

### Animals, diets, and reagents

All animal care and handling procedures were approved by the Institutional Animal Care and Use Committee of Osaka University. Mice were housed in a temperature-, humidity-, and light-controlled room with a 12-h light/12-h dark cycle (lights on at 08:00 a.m.) and allowed free access to water and chow. Mating, genotyping, and feeding procedures were as previously described (Sakurai et al., 2012). In brief, F_1_ mice (+/+, PACAP/+, A^y^/+, and A^y^/+:PACAP/+) were obtained by mating female transgenic mice overexpressing PACAP in their pancreatic β cells (Yamamoto et al., 2003) with male KKA^y^ mice (KK-A^y^/Ta mice, CLEA Japan Inc., Tokyo, Japan). F_1_ males were individually housed after genotyping and weaning, and males with the A^y^ allele (A^y^/+ and A^y^/+:PACAP/+) were fed with an HFD (HFD-32, CLEA Japan, Tokyo, Japan) from 4 weeks of age, while the other males continued on a normal diet (ND) (DC-8, CLEA Japan). These diets contain either 11.8% (DC-8) or 56.7% (HFD-32) of energy derived from fat.

### Histochemistry

From each deeply anesthetized mouse, the pancreas was removed, weighed, and immediately fixed in 4% paraformaldehyde in phosphate buffered saline solution. Samples were embedded in paraffin, and 5 μm sections were prepared for HE or AF staining, or immunohistochemical staining with anti-insulin (N1542, DAKO, Carpinteria, CA, USA) or anti-glucagon (N1541, DAKO) antibodies, in which signals were visualized using the diaminobenzidine method and were counterstained with cresyl violet. To investigate the architectural changes in the islets, two serial sections were prepared for immunostaining with anti-insulin and anti-glucagon antibodies, respectively.

### Morphometry

Stained sections were photographed using a BIOREVO BZ-9000 microscope (Keyence, Japan), and morphometrical parameters were examined as follows. In HE-stained sections (*n* = 4–6 for 5-month-old mice, and *n* = 7–9 for 11-month-old mice), total islet number, size of each islet, and total pancreatic area were counted or measured, and analyzed as previously described (Tomimoto et al., 2004). Briefly, in each section from four F_1_ groups, the mean islet size was determined by averaging the size of each islet, and the islet density by dividing total islet number by total pancreatic area (mm^2^). Islet mass was calculated by multiplying the pancreas weight by the relative islet area per pancreas. The density per mm^2^ of total pancreatic area, in addition to the frequency of the six groups of optical islet size (<0.003, 0.003–0.01, 0.01–0.03, 0.03–0.1, 0.1–0.3, and >0.3 mm^2^), was also determined. In AF-stained sections (*n* = 5 for 5-month-old mice, and *n* = 3 for 11-month-old mice), blinded observers evaluated the AF reagent staining in each islet of four F_1_ groups. In sections stained with anti-insulin or anti-glucagon antibodies (*n* = 4 for each group), the positive area and the size of each insulin-positive cluster were measured using ImageJ software (version 1.30, http://rsb.info.nih.gov/ij). The number of insulin-positive clusters, and of glucagon cell-infiltrated islets (which exhibit glucagon-positive cells inside of islets as indicated by arrows in Figure 2B), were also counted and analyzed.

### Plasma insulin level in an intraperitoneal glucose tolerance test

Two milligrams per kilogram glucose was intraperitoneally injected to each mouse after a 14-h food deprivation as described (Sakurai et al., 2012). Plasma samples were prepared just before (time 0) and at 10, 30, 60, 90, and 120 min after glucose load, and the insulin level in the samples was examined by a mouse insulin enzyme-linked immunosorbent assay (Morinaga, Tokyo, Japan).

### Data analysis and statistics

All data are expressed as mean ± standard error of the mean. Statistical evaluation was carried out using KaleidaGraph software (HULINKS, Tokyo, Japan). The statistical significance of differences was assessed using two-way ANOVA followed by the Tukey–Kramer test, χ^2^ test, and Student’s un-paired *t*-test. Differences with *P* < 0.05 were considered significant.

## Results

### Effects on size and density of islets

**Figure 1:**
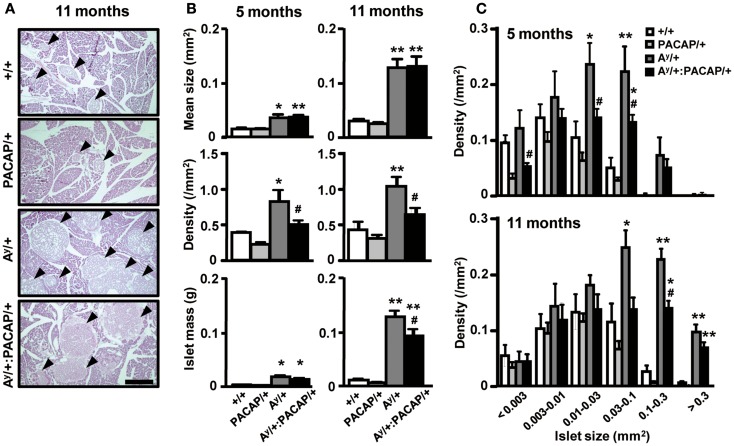
**Quantitative islet histomorphometry of hematoxylin-eosin-stained pancreatic sections of 5- and 11-month-old F_1_ mice**. **(A)** Representative images of pancreatic sections of +/+ (wild-type), PACAP/+, A^y^/+, and PACAP/+:A^y^/+ mice stained with hematoxylin-and-eosin (HE). Arrow heads indicate islets. Scale bar, 500 μm. **(B,C)** Morphometric data of the HE-stained pancreatic sections. F_1_ male littermates of 5-month-old (*n* = 4–6) and 11-month-old (*n* = 7–9) mice were examined. **(B)** Mean islet size, density of islets (the number of islets per square millimeter of total pancreatic area), and the calculated total islet mass in F_1_ mice. **(C)** Density of islets of the indicated size in F_1_ mice. Data are expressed as the mean + SEM. *P < 0.05, **P < 0.01, versus representative control with or without PACAP, ^#^*P* < 0.05, versus Ay/+ mice, one-way ANOVA followed by the Tukey–Kramer test.

In KKA^y^ mice fed with a ND, we previously showed that pancreatic PACAP overexpression markedly suppresses the increase of mean islet size and of islet density (Tomimoto et al., 2004). Contrary to these observations, HE-stained pancreatic sections from 11-month-old HFD-fed KKA^y^ mice showed that not only A^y^/+ mice, but also A^y^/+:PACAP/+ mice, exhibited a clear islet enlargement (Figure 1A). Quantitative analyses indicated that the mean size was significantly increased in both groups compared with their respective controls, but no significant difference was observed between A^y^/+ and A^y^/+:PACAP/+ mice at either 5 or 11 months of age (Figure 1B, upper graphs). In contrast, a significant increase in inlet density was observed in A^y^/+ but not in A^y^/+:PACAP/+ mice, and the density in A^y^/+:PACAP/+ mice was significantly suppressed compared with A^y^/+ mice at both 5 and 11 months of age (Figure 1B, middle graphs). With respect to islet mass, there was a large increase in A^y^/+ mice (e.g., it was 22-fold higher than +/+ mice at 11 months of age; Figure 1B, lower graphs). In a model showing such remarkable islet mass expansion, pancreatic PACAP overexpression caused a 30% reduction in islet mass at 11 months of age (Figure 1B, lower right graph). Taken together with our previous results in ND-fed KKA^y^ mice (Tomimoto et al., 2004), these results indicate that the inhibitory effects of PACAP on islet density are retained, but those influencing islet size are lost, in HFD-fed KKA^y^ mice. Note that no significant differences were observed between +/+ and PACAP/+ mice for any of the parameters measured, as described (Tomimoto et al., 2004).

### Effects on islets of various sizes

We next examined which size of islets was decreased by PACAP in HFD-fed KKA^y^ mice (Figure 1C). In both 5- and 11-month-old groups, the density of larger (>0.03 mm^2^) islets was preferentially increased in A^y^/+ mice compared with +/+ mice, and this increase was significantly inhibited in A^y^/+:PACAP/+ mice. Since the density of smaller (<0.003 mm^2^) islets was also decreased in A^y^/+:PACAP/+ mice at 5 months of age, we also compared the density of islets sized <0.001 mm^2^ between A^y^/+ and A^y^/+:PACAP/+ mice at that age (Table 1). The result showed an 80% decrease in the density of these tiny islets in A^y^/+:PACAP/+ mice, supporting the observation that a marked decrease in the smaller islets has indeed occurred in A^y^/+:PACAP/+ mice. In contrast, size distribution analysis indicated that the distribution in A^y^/+:PACAP/+ mice was almost the same as A^y^/+ mice at both ages (5 months old, χ^2^ = 2.33, *P* = 0.802; 11 months old, χ^2^ = 1.33, *P* = 0.932), whereas they were clearly shifted right compared to +/+ mice (for example, in A^y^/+ versus +/+ mice; 5 months old, χ^2^ = 19.0, *P* < 0.01; 11 months old, χ^2^ = 35.0, *P* < 0.0001). Collectively, these results suggest that PACAP overexpression does not affect the overall size distribution of islets in HFD-KKA^y^ mice, but has clear inhibitory effects on islet density, particularly in the smaller islets (<0.003 mm^2^) at an earlier age (5 months old).

**Table 1:** **Parameters of islets in A^y^/+ and A^y^/+:PACAP/+ mice fed a high-fat diet**.

Morphological parameters	A^y^/+	A^y^/+:PACAP/+	*P*-value
*HE-stained sections*			
Density of tiny islets* (/mm^2^)	2.00 ± 0.32	0.40 ± 0.25	0.004^†^
*Insulin-immunostained sections*			
Insulin-positive area (% of sections)	4.17 ± 1.07	2.28 ± 0.18	0.127
Density of insulin clusters (/mm^2^)	1.21 ± 0.16	0.92 ± 0.08	0.159
Frequency of ductal clusters (%)	9.81 ± 0.84	5.88 ± 1.75	0.140
Density of ductal clusters (/mm^2^)	0.12 ± 0.02	0.05 ± 0.01	0.048^†^
Mean size of insulin clusters (μm^2^)	32.6 ± 4.5	25.1 ± 2.2	0.189
*Glucagon-immunostained sections*			
Glucagon-positive area (% of sections)	0.46 ± 0.07	0.49 ± 0.11	0.787
Infiltrated islets^#^ (% of islets)	15.4 ± 2.8	21.3 ± 2.2	0.160

Pancreatic sections prepared from 5-month-old A^y^/+ and A^y^/+:PACAP/+ mice were stained with hematoxylin-and-eosin (HE, *n* = 5 for each group), anti-insulin antibodies, or anti-glucagon antibodies (*n* = 4 for each group), and were subjected to morphometric analyses. Statistical analyses were performed using an un-paired Student’s *t*-test. ^†^Values regarded as statistically significant. *Tiny islets are those of area <0.001 mm^2^. ^#^Infiltrated islets are those that have glucagon-positive cells inside, in addition to at their periphery.

### Observation of AF-stained sections

**Figure 2:**
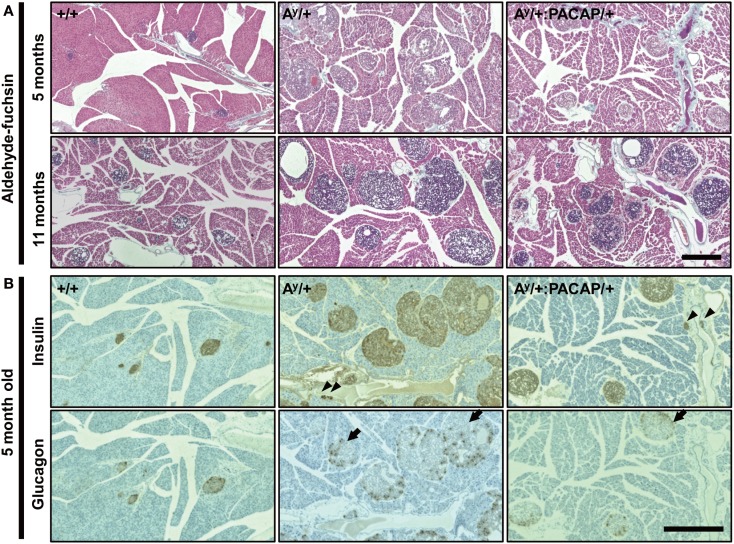
**Comparative histology of aldehyde-fuchsin, and anti-insulin or anti-glucagon-stained pancreatic sections of F_1_ mice**. **(A)** Pancreatic sections from 5 to 11 months old +/+ (wild-type), A^y^/+, and PACAP/+:A^y^/+ mice were stained with aldehyde-fuchsin (AF) reagent to detect granulated β-cells. Note that a lack of AF staining (degranulation of β-cells) is observed in islets of A^y^/+ and PACAP/+:A^y^/+ mice but not in +/+ mice at 5 months old, but clear staining is generally seen in 11-month-old animals. **(B)** Two adjacent pancreatic sections from 5-month-old +/+, A^y^/+, and PACAP/+:A^y^/+ mice were stained with anti-insulin and anti-glucagon antibodies, respectively. Arrowheads indicate ductal insulin-positive clusters, whereas arrows denote glucagon cell-infiltrated islets in which glucagon-positive cells reside inside in addition to at the periphery of the islet. Note both types of signals were rarely observed in +/+ mice. Scale bars, 500 μm.

To explore the possible changes of islets in A^y^/+ and A^y^/+:PACAP/+ mice, we used AF staining to examine the degranulation of β-cells, a well known phenotypic change in obese mouse islets, including those of KKA^y^ mice (Iwatsuka et al., 1970). As shown in Figure 2A, we observed a clear disappearance of AF staining in HFD-fed A^y^/+ mice, but not in +/+ mice, at 5 months of age. However, we unexpectedly observed that AF staining was obvious in islets of 11-month-old A^y^/+ mice, implying that a compensatory reaction had occurred. When comparing between A^y^/+ and A^y^/+:PACAP/+ mice, all islets in all samples at 5 months of age (*n* = 5 for each genotypes) lacked AF staining, whereas those at 11 months of age (*n* = 3 for each genotype) showed definite AF staining. These results suggest that an unexpected recovery in β-cell degranulation could be occurring in HFD-fed KKA^y^ mice, and that pancreatic PACAP overexpression does not affect either degranulation, or compensatory re-granulation, of β-cells in this model.

### Intraperitoneal glucose tolerance test

**Figure 3:**
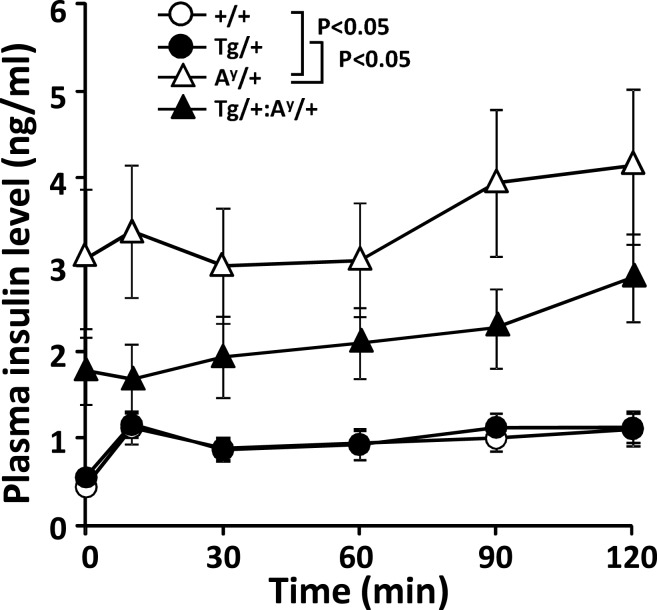
**Changes in plasma insulin levels in an intraperitoneal glucose tolerance test (ipGTT) of 11-month-old F_1_ mice**. Eleven-month-old +/+ (wild-type, *n* = 13), PACAP/+ (*n* = 20), A^y^/+ (*n* = 20), and PACAP/+:A^y^/+ (*n* = 27) mice were subjected to ipGTT (Sakurai et al., 2012). Insulin concentration of each plasma sample was determined. *P < 0.05 between the indicated groups (two-way repeated-measures ANOVA).

In line with the unexpected recovery in β-cell degranulation, we previously showed that the glucose disposal in HFD-fed KKA^y^ mice is unexpectedly enhanced at 11 months of age compared with their age-matched wild-types (Sakurai et al., 2012). Therefore, we here checked the glucose-induced elevation of plasma insulin level in the 11-month-old F_1_ groups (Figure 3). The results indicated that the insulin level in A^y^/+ but not A^y^/+:PACAP/+ mice is significantly elevated compared with +/+ and PACAP/+ mice even under fasted state. On the other hand, the first-phase insulin response (value dividing the insulin level at 10 min by that at 0 min) was attenuated in both of A^y^/+ and A^y^/+:PACAP/+ compared with wild-type mice, but no significant difference was observed between two groups (The first-phase insulin response: +/+, 2.73 ± 0.46; PACAP/+, 2.72 ± 0.35; A^y^/+, 1.16 ± 0.11; A^y^/+:PACAP/+, 1.22 ± 0.26). These data suggest that the impaired first-phase insulin response seems to be persistently observed in both A^y^/+ and A^y^/+:PACAP/+ mice, and that pancreatic PACAP overexpression showed little effects on the glucose-induced insulin release at least at 11 months of age.

### Effects on insulin or glucagon cells

The above data indicate that phenotypic differences between the 5-month-old groups were more obvious and are possibly causative for the changes in 11-month-old groups. Thus, we next performed detailed analyses on the islets of 5-month-old A^y^/+ and PACAP/+:A^y^/+ mice using two adjacent sections stained with anti-insulin and anti-glucagon antibodies, respectively (Figure 2B). In insulin-stained section, well-stained islets were commonly observed in both A^y^/+ and PACAP/+:A^y^/+ mice. Compared with +/+ mice, not only enlarged islets but also small insulin-positive clusters, some of which were located in the ductal structures (as indicated by arrowheads), were often observed in these two groups. Glucagon-stained sections revealed an apparent reduction of staining in both A^y^/+ and PACAP/+:A^y^/+ mice compared with +/+ mice. In addition, some islets showed an altered localization of glucagon-positive cells (as indicated by arrows); namely, the cells localized not only peripherally but also in the central area of the islets. In age-matched +/+ mice, this was rarely observed and glucagon-positive cells were predominantly localized at the periphery of the islets. Based on these observations, a range of parameters were examined (Table 1). In insulin-stained sections, quantitative analysis revealed a tendency toward a decrease, but no significant change, in the percent of insulin-positive area, the density, and the mean size of insulin-positive clusters between A^y^/+ and A^y^/+:PACAP/+ mice. However, the density of ductal insulin-positive clusters was significantly decreased (by 58%) in A^y^/+:PACAP/+ mice compared with A^y^/+ mice, whereas the total frequency of ductal clusters was not significantly different. In glucagon-stained sections, no significant difference was observed in the percent of glucagon-positive area and of glucagon cell-infiltrated islets.

## Discussion

We examined the morphoregulative roles of PACAP on pancreatic islets using a severe and early-onset type II diabetes model (HFD-fed KKA^y^ mice; Sakurai et al., 2012) over the course of approximately 1 year. In A^y^/+:PACAP/+ mice, the significant suppression of islet mass expansion at 11 but not at 5 months of age (Figure 1B) fits with previous data showing attenuation of enhanced hyperinsulinemia during 6–10 months of age (Sakurai et al., 2012). In contrast, between the age-matched A^y^/+ and A^y^/+:PACAP/+ mice, other morphometric analyses revealed no significant difference in β-cell granulation, insulin-, and the glucagon-positive area per pancreatic section, or the distribution of glucagon-positive cells in islets (Figure 2; Table 1). In addition, the data in intraperitoneal glucose tolerance test (ipGTT) also suggest that no significant effects of PACAP on the glucose-induced insulin release. These results therefore suggest that PACAP inhibits morphological rather than functional changes in islets and thereby suppresses the increase of hyperinsulinemia in A^y^/+:PACAP/+ mice. In addition, taken together with results in ND-fed KKA^y^ mice (Tomimoto et al., 2004), the present study provides additional evidence showing morphoregulative roles of PACAP on pancreatic islets, and establishes the inhibitory effects of PACAP on compensatory islet mass expansion in type II diabetes.

The present study revealed sustained suppression (at least between 5 and 11 months of age) of islet density, but not of mean islet size, in A^y^/+:PACAP/+ mice compared with A^y^/+ mice (Figure 1B). Taken together with our previous results (Tomimoto et al., 2004), these data indicate that PACAP universally and persistently inhibits the increase of islet density from the early postnatal period in type II diabetes models. It is unlikely that HFD and A^y^ allele-boosted islet enlargement masked PACAP’s inhibitory effects on mean islet size, because the mean islet size in 5-month-old A^y^/+ mice (0.35 ± 0.06 mm^2^) was less than half of that observed in ND-fed KKA^y^ mice (0.80 ± 0.08 mm^2^; Tomimoto et al., 2004). Size-fractionated analyses indicated that the increase of larger-sized islets in A^y^/+ mice is preferentially suppressed by PACAP at both 5 and 11 months, but also revealed a large decrease in the smaller islets in A^y^/+:PACAP/+ mice at 5 months of age (Figure 1C; Table 1). These data are consistent with PACAP’s effects at earlier ages, and suggest PACAP-induced reduction of small-sized islets as a core phenotype of A^y^/+:PACAP/+ mice, which eventually contributes to the decrease of larger islets and of islet mass in these mice compared with A^y^/+ mice.

In A^y^/+:PACAP/+ mice fed with ND, we previously showed that the pancreatic PACAP content and the plasma insulin level are increased by 3.5- and 2.8-fold of PACAP/+ mice (Tomimoto et al., 2004), suggesting that A^y^ allele-related increase in the plasma insulin boosts the PACAP expression in PACAP/+ mice by activating the human insulin promoter cassette of the transgene construct (Yamamoto et al., 2003). Although we did not checked the PACAP content in the present study, the pancreatic content in HFD-fed A^y^/+:PACAP/+ mice could be estimated as more than 10-fold compared with +/+ mice, because their plasma insulin level is 70- to 100-fold compared with +/+ mice (Sakurai et al., 2012) whereas ND-fed A^y^/+:PACAP/+ mice showed 10-fold increase in PACAP with 2.8-fold increase in plasma insulin compared with +/+ mice (Tomimoto et al., 2004). Since our previous study showed that the pancreatic PACAP content is 48 ± 12 pg/mg (Tomimoto et al., 2004), it could be translated that approximately 10 pM PACAP exists in pancreas or 1 nM PACAP locally exists around islets (because PACAP is known to be produced only nearby islets that corresponds to 1% volume of pancreas). Therefore, if the PACAP content was increased by more than 10-fold in HFD-fed A^y^/+:PACAP/+ mice, it could be activate various intracellular signaling pathway, because higher concentration of PACAP is known to stimulate Gq-linked pathway in addition to Gs-linked pathway via binding to PAC1, VPAC1, and VPAC2 receptors (Vaudry et al., 2009). Thus, it should be noted that further studies are required to determine the molecular mechanism underlying the phenotypic changes observed.

Based on current knowledge, the number of small islets (β-cell clusters) is regulated via diverse processes including differentiation from ductal precursor cells, trans-differentiation from non-β-cells, fission or fusion between islets, and replication, hypertrophy, and apoptosis of the β-cells themselves (Bonner-Weir et al., 2004; Brennand and Melton, 2009). Although further studies on how these processes may be affected by PACAP should be performed, the significant decrease in the density of ductal insulin-positive clusters in the context of a normal frequency of these clusters in the duct (Table 1) strongly suggests that PACAP inhibits ductal precursor-related islet neogenesis in A^y^/+:PACAP/+ mice. If this is the case, it is likely that differentiation from precursor cells, and/or the cell-fate regulation of newly produced β-cells from these precursors, are the possible causative mechanisms explaining PACAP-induced reduction of small-sized islets in A^y^/+:PACAP/+ mice.

In conclusion, the present study provides additional evidence for the inhibitory effects of PACAP on pancreatic β-cell mass expansion, and suggested its possible effects on ductal precursor-related islet genesis. An increased number of ductal insulin-positive cells has been reported in pancreatic biopsy samples from human type II diabetic patients, in which the increased β-cell mass was suggested to be due to increased islet neogenesis but not to islet enlargement (Butler et al., 2003). Since these results imply that the regulation of islet mass depends on species or disease state-dependent differences, future studies on (postnatal) islet neogenesis are important for a deeper understanding of islet homeostasis in type II diabetes, because a number of unrelated and sometimes contradictory results appear to have accumulated in this research field.

## Conflict of Interest Statement

The authors declare that the research was conducted in the absence of any commercial or financial relationships that could be construed as a potential conflict of interest.
